# RECAPDOC - a questionnaire for the documentation of rehabilitation care utilization in individuals with disorders of consciousness in long-term care in Germany: development and pretesting

**DOI:** 10.1186/s12913-018-3153-3

**Published:** 2018-05-04

**Authors:** Hanna Klingshirn, Rene Mittrach, Kathrin Braitmayer, Ralf Strobl, Andreas Bender, Eva Grill, Martin Müller

**Affiliations:** 1 Institute of Medical Information Processing, Biometry and Epidemiology, Ludwig-Maximilians-Universität München, Marchioninistr. 17, 81377 München, Germany; 2 German Center for Vertigo and Balance Disorders, Ludwig-Maximilians-Universität München, Marchioninistr. 15, 81377 München, Germany; 3 Department of Neurology, Ludwig-Maximilians-Universität München, Marchioninistr. 15, 81377 München, Germany; 4 Therapiezentrum Burgau, Kapuzinerstraße 34, 89331 Burgau, Germany; 5 Faculty of Applied Health and Social Sciences, Rosenheim University of Applied Sciences, Hochschulstr.1, 83024 Rosenheim, Germany

**Keywords:** Questionnaire development, Cognitive interview, Pretest, Rehabilitation, Long-term care, Disorder of consciousness, Vegetative state, Minimally conscious state

## Abstract

**Background:**

A multitude of different rehabilitation interventions and other specific health care services are offered for individuals with disorders of consciousness in long-term care settings. To investigate the association of those services and patient-relevant outcomes, a specific instrument to document the utilization of those services is needed. The purpose of this study was to develop such a questionnaire administered to caregivers in epidemiological studies or patient registries in Germany.

**Methods:**

The development process of the RECAPDOC questionnaire was carried out in three steps. Step 1 consisted of a systematic literature review and an online-based expert survey to define the general content. Step 2 was an expert interview to evaluate the preliminary content of the questionnaire. Step 3 was a pretest including cognitive interviews with caregivers. After each step, the results were combined into a new version of the questionnaire.

**Results:**

The first version of the questionnaire included items on utilization of medical care, medical aids, nursing and therapeutic care. The results of the expert interview led to the integration of five new items and the modification of six other items. The pretest led to some minor modifications of the questionnaire since it was rated as feasible and acceptable. The final questionnaire consisted of 29 items covering the domains “living situation”, “social insurance status”, “utilisation of home health care”, “domestic services”, “outpatient health care”, “specific diagnostic measures”, “adaptive technologies”, “medical aids” and “utilization of therapies”. Also the experience of family support and multidisciplinary collaboration of health professionals is covered.

**Conclusions:**

The developed questionnaire is a first step to make the situation of patients with disorders of consciousness in the long-term care setting accessible for evaluation in epidemiological studies and in the context of patient registries. However, further reliability and validity studies are needed.

**Electronic supplementary material:**

The online version of this article (10.1186/s12913-018-3153-3) contains supplementary material, which is available to authorized users.

## Background

More and more individuals survive severe brain injuries due to optimized treatment in the acute and the post-acute situation. Consequently, the number of those with chronic disorders of consciousness (DOC) increases [1]. DOC is characterized as coma (complete unawareness, eyes-closed state), vegetative state (VS; complete unresponsive, eyes-open state), or unresponsive wakefulness syndrome (UWS, as a more neutral and descriptive term) and minimally conscious state (MCS; limited conscious interaction with the environment) [2, 3].

Despite the presumably increasing number of individuals with DOC, current knowledge on diagnosis, optimal management and treatment is scarce. Furthermore, reliable data on prevalence and incidence is limited and shows great variation [4, 5]. For examestimation of VS ranges from 2.8 to 3.4 and for MCS from 1.5 to 2.8 per 100,000 [6, 7].

A major challenge in management of individuals with DOC is the prediction of long-term outcomes. For the last two decades, VS was considered as permanent when lasting longer than 3 month in individuals with non-traumatic brain injuries or 12 months in those with traumatic brain injuries [8, 9]. However, recent studies with long-term follow-up showed that recovery of consciousness and functional progress is possible, even 2 to 5 years post injury [10–15]. A systematic review from 2015 revealed that modern diagnostic methods are still not satisfactory regarding reliability of diagnoses and prognosis [16] indicating that decisions regarding treatment and rehabilitation withdrawal are far from evidence-based. Also, findings from a German prospective multicentre neurologic rehabilitation registry support these findings and indicate that clinical improvement may take place even several months after brain injury [17–19], despite poor initial prognosis. Considering this, it seems advisable, that a proper level of rehabilitation care has to be maintained even after discharge from initial inpatient rehabilitation and the effectiveness of offered rehabilitation and other health care interventions has to be evaluated carefully. A systematic review of rehabilitation interventions in the long term care setting has shown that implemented interventions are very diverse, their quality of evidence is low and high quality research is needed [20]. Epidemiological research and research in patient registries needs instruments to collect data on potential determinants of favourable patient-relevant outcomes. However, instruments that are able to reflect the specific pattern of health care utilization in individuals with DOC and that can be used alongside more general instruments on health care utilization are missing so far. Therefore, the objective of this study was to develop a specific questionnaire to document provision of health care services in individuals with DOC living in an inpatient or outpatient long-term care setting for the use in epidemiological studies or patient registries.

## Methods

**Fig. 1:**
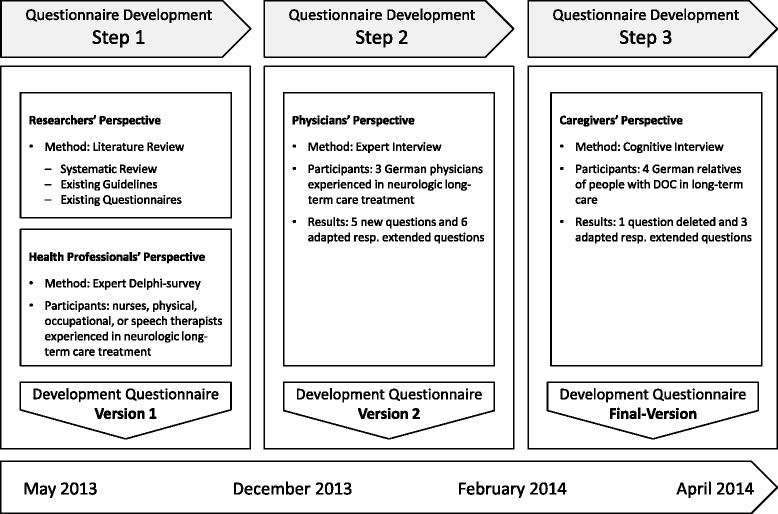
Development process of the questionnaire to investigate rehabilitation care of individuals with disorders of consciousness (DOC) in long-term care

The development process of the RECAPDOC questionnaire was carried out in three subsequent steps and aimed to take into account all relevant perspectives, i.e. those of researchers in the field, health professionals, physicians, and lay caregivers (see Fig. 1). First, a systematic review of the literature, a review of existing guidelines and a review of existing questionnaires for health resource utilization as well as online Delphi-survey with clinical experts were carried out to determine the content of the questionnaire (Step 1). These results were summarized in a first version of the questionnaire. Second, we carried out an expert interview with physicians specialized in the treatment and management of individuals with DOC to define specific content relevant from the physician’s perspective (Step2). These results were summarized in a second version of the questionnaire. Finally, a pretest with cognitive interviews was carried out to evaluate feasibility and comprehensibility of the questionnaire (Step 3). This step led to a final version of the questionnaire.

### Researchers’ perspective: literature review

#### Systematic review

The full description of the methods of the systematic review can be found elsewhere [20]. In brief, the objective of the systematic review was to identify rehabilitation interventions for individuals with DOC in long term care.

#### Existing guidelines

To design an instrument that is in line with current health care practice, a comprehensive online and hand search was carried out to identify relevant practice guidelines or recommendations from professional organisations.

#### Existing questionnaires

To build our questionnaire upon the experience of related work and make is useable together with instruments that are more generic, existing questionnaires for health resource utilization in Germany were reviewed.

### Health professionals’ perspective: delphi-survey

The objective of the expert Delphi-survey was to substantiate the content of the questionnaire in terms of describing the current practice of rehabilitation care of health professionals in the long-term care setting in individuals with DOC in Germany. Experts were defined as health care professionals (nurses, physical therapists, occupational therapists or speech and language therapists) with at least 5 years of professional experience in rehabilitation of individuals with DOC, advanced specialized education in the field of neurological rehabilitation and experience in the long-term care setting.

**Fig. 2:**
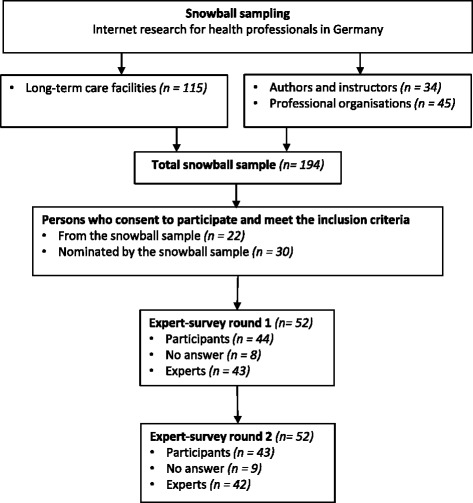
Flow of participants of expert Delphi-survey

Experts were recruited via snowball sampling. We contacted national long-term care facilities, authors of relevant publications, scholars from nursing and rehabilitation sciences, and national professional organisations and asked to nominate experts according the definition (see Fig. 2).

All recruited experts were asked to participate in two rounds of an internet-based Delphi survey [21].

In round one (July 2013), the participants were approached with questions on the use of special treatment concepts, adaptive technologies and medical aids, measures to support the family, measures to support social participation and measures to support interdisciplinary collaborations using open-ended questions. In round two (September 2013), each answer that was named more than once in round one was presented to the same group of experts in closed-ended questions and the experts were asked to rate the relevance of each item. As an example, experts were asked to rate the relevance of a certain therapeutic approach for patients with DOC in the targeted setting on a four-point Likert scale (“very important”, “important”, “slightly important”, “not important at all”). Every category that was rated as “very important” or “important” by a majority of the experts (> 50%) was included in the questionnaire. To describe the participating experts, each round contained questions about age, sex and profession and inclusion criteria (see Additional file 1).

### Physicians’ perspective: expert interview

According to the guidelines of the German Federal Rehabilitation Council and the funding agencies of the German Federal Health Insurance [22] rehabilitation in long-term care setting has to be coordinated by a specialized physician. To define content relevant from the physician’s perspective, the questionnaire was evaluated via semi-structured telephone interviews with physicians specialized in neurology and expertise in rehabilitation in the long-term care context.

The physicians were recruited from cooperating clinics in different federals states of Germany and following personal suggestions. Sample size was determined by saturation, i.e. the point where new data collection is unlikely to provide new insights [23]. The current version of the questionnaire was sent to the participants 5 days before the interview. During the telephone interview, the participants were asked to comment of the relevance and comprehensibility of items related to medical care, i.e. items on consultation of medical specialists, use of specialized treatment concepts, adaptive technologies and medical aids, or therapeutic services (see Additional file 2).

The interviews were documented in written form and analysed via investigator triangulation involving two independent researchers. This should minimize the influence of an individual researcher and lead to a higher level of reliability of the analysis [24].

### Caregivers’ perspective: cognitive interviews

To assess feasibility and comprehensibility of the RECAPDOC questionnaire, semi-structured cognitive interviews [25–27] with caregivers were carried out. Sample size was determined by saturation, i.e. participants were included up to the point were inclusion of new participants is unlikely to provide new insights [23]. The caregivers were recruited via the German prospective multicentre neurologic rehabilitation registry [18]. This registry was set up in five facilities across the state of Bavaria/Germany with a special expertise in the rehabilitation of acquired brain injury and was started in August 2011. A staff member of the registry contacted the caregivers and asked for consent to be contacted for the study. If the caregivers consented, members of the research team scheduled an interview. Inclusion criteria were: the individuals cared for receive long-term care (in Phase F, according to the classification of neurological rehabilitation by the German Statutory Pension Insurance Scheme), and diagnosis was either coma, VS or MCS. When necessary, a declaration of consent of the legal guardian of the patient had been obtained prior to the start of the interviews. To meet the participants’ needs and to minimize their burden, all interviews took place in the participants’ homes.

During the interview process, the techniques ‘think-aloud’, ‘probing’ and ‘observation of respondents behaviour’ were used [26, 27]. This means that we asked the participants to verbally express their thoughts during filling each item of the questionnaire. We also requested to mention any ambiguities or other comprehension problems. In the case that items were answered without any verbal statement, the participants were reminded of the initial instructions and the interviewers asked further questions to clarify the thoughts of the participant. In addition, the participants’ behaviour was observed and interesting observations like flipping a page back, entry at the wrong position in the questionnaire or facial expressions were documented and addressed immediately. Additional questions (‘probes’) were asked to investigate whether the questionnaire items were understood correctly.

After completing the questionnaire, the method of ‘confidence rating’ was used to evaluate the reliability of the answers (e.g. on duration of therapies or expenses). The participants were asked to rate their confidence in their own answers using a numerical scale ranging from 1 to 10, where 1 means absolutely no confidence in the answer and 10 means a maximum confidence. The same scale was used to assess the general feasibility of the questionnaire and to rate the usefulness of the instructions.

All interviews were conducted and recorded after the participant had been properly informed and given their written consent. The interviews were transcribed verbatim and complemented with field notes. The results were evaluated using investigator triangulation [24] involving two independent researchers.

After each step, potential changes in the questionnaire were discussed among all involved members of the research team until consent was reached.

## Results

### Researchers’ perspective: literature review

#### Systematic review

The detailed results of the systematic review on rehabilitation interventions for patients with DOC in long-term care are described elsewhere [20]. In brief, this systematic review could not identify any effective rehabilitation intervention, but gave an overview of current clinical practice. A list of categories of rehabilitation interventions was developed from the included studies and defined the first content of the questionnaire.

#### Existing guidelines

The search identified one relevant guideline, the “Recommendations for inpatient long-term care and treatment of people with severe brain damage in the phase F” provided by the major federal funding bodies of rehabilitation care, the German Federal Rehabilitation Council [22]. This guideline describes interventions provided by nurses, physical, occupational, and speech and language therapists, the use of special treatment concepts, interdisciplinary collaborations, environmental adaptions e.g. adaptive technologies and medical aids and measures to support the family and participation in community as essential components of rehabilitation care for individuals with severe brain injuries. Since the guideline emphasizes the involvement of the personal caregivers in all therapies as well as intensive counselling, we developed a scale to document the implementation of this issue (see Table 1).

**Table 1:** Scale to document the involvement of personal caregivers in therapies and care (to be documented for each profession separately)

	Fully applies	Partly applies	Does rather not apply	Does not apply at all
I feel adequately informed.				
I feel consulted by the therapist/nurse in a competent way.				
I feel well guided to continuously implement therapeutic/ nursing measures.				
I feel fully involved into the therapeutic process.				
I feel physical and psychological relief through the therapy.				
I feel supported to realize an appropriate extent of social participation for the patient.				

#### Existing questionnaires

The search identified the “German questionnaire for health-related resource use in the elderly population – FIMA” [28] as the only relevant instrument. In order to use this experience and to facilitate a joint use, we decided to use its time frames (3 months retrospectively), question formats, response options and general layout. Permission and further consultation was obtained from the first author of the questionnaire.

The synthesis of the result of the systematic review, the guideline search and the search for existing questionnaires resulted in an initial version of the questionnaire.

### Health professionals’ perspective: delphi survey

Fifty-two national experts (from 194 initially contacted, see Fig. 2) agreed to participate in the Delphi-survey. A total of 43 experts participated in round one and 42 in round two (see Fig. 2). Characteristics of experts participated in round two are shown in Table 2.

**Table 2:** Characteristics of the 42 participants from the expert survey

	Percent	Number
Sex^a^		
Female	66	(27)
Male	34	(14)
Profession		
Nurse	36	(15)
Physical therapist	19	(8)
Occupational therapist	12	(5)
Speech and language therapist	26	(11)
Other therapeutic	7	(3)
Experience in outpatient long-term care (years)	62	(26)
Experience in neurologic day-care centre (years)	17	(7)
Experience in nursing home (years)	45	(19)
Experience in special neurologic nursing home(years)	64	(27)
	M (SD)	Min-Max
Age (years)^b^	47 (8)	30–62
Professional experience in neurologic rehabilitation (years)	18 (9)	5–43
Professional experience in neurologic long-term care (years)	13 (7)	0.5–35

^a^Information on sex was not provided by 1 participant

^b^Information on age was not provided by 5 participants

In round one, experts named 23 items that could be classified into six topics (*special treatment concepts*, *adaptive technologies and medical aids*, *measures to support the family*, *measures to support social participation* and *measures to support interdisciplinary collaborations*). In round two, 17 items from round one were judged to be relevant (i.e. was rated as “very important” or “important” by at least 50%). In the topic “special treatment concepts”, four out of the 10 suggested concepts were not judged as relevant and where therefore excluded from the questionnaire (see Table 3).

**Table 3:** Results of the expert Delphi survey

Topic	Topic-related items judged as relevant by the expert participants
Special treatment concepts	Facio-Oral Tract Therapy (FOTT), Stimulation programs, Neuro-developmental treatment (Bobath therapy), Affolter therapy, Kinaestetics
Adaptive technologies and medical aids	Wheel chairs, positioning materials, suction units, hospital beds, tracheostomy equipment, bathroom hoists and seats, standing boards, communication devices, mobility devices, orthotics/ splints
Measures to support the family	Providing information, consulting and counselling, teaching, integration into nursing/therapy process, physical help and emotional relief
Goals of successful rehabilitation	Communication, perception, mobility, interaction and social relationships, self- sufficiency and social participation
Measures to support interdisciplinary collaborations	Interdisciplinary care plans and documentation, regular team meetings and case conferences, conceptual alignment, inter-professional practice

The results of the Delphi survey and the literature review were combined into the version 1 of the RECAPDOC questionnaire (see Fig. 1).

### Physicians’ perspective: expert interview

Three medical directors of neurological rehabilitation facilities from different German regions experienced in long-term care treatment of individuals with DOC were recruited for the interviews. Those experts suggested the integration of five new items into the questionnaire: *Evaluation by electroencephalogram*, *main diagnosis*, *date of injury*, *discharge from acute rehabilitation* and *payments from the German social accident insurance*. In addition, they proposed small adaptations of another six items on living conditions, level of care, number of physician visits, medical aids, treatment concepts and medical treatments.

The resulting changes led to version 2 of the RECAPDOC questionnaire.

### Caregivers’ perspective: cognitive interview

We contacted the legal guardians of 25 affected individuals. Among those, eight persons could not be reached. Finally, four personal caregivers – which were also legal guardians - agreed to participate in the interviews. Three interviews took place in the participants’ private homes and one was conducted in a nursing home.

On average, it took the participants 17 min to complete the questionnaire. All participants rated the questionnaire as easy to manage and comprehensible, except the items referring to the special treatment concepts. Participants rated their responses on time intervals and financial resources as being very accurate.

The findings from the cognitive interviews led to removing the items regarding special treatment concepts. In addition, some minor adaptions were necessary. First, the item regarding the level of care was amended by a detailed explanation of the term “hardship provision”. Second, the item on involvement of a multidisciplinary rehabilitation team was rephrased with less abstract terms. Third, the item on utilization of adaptive technologies and medical aids was amended by an explanation of the ownership status, e.g. owning a wheelchair does not necessarily require its purchase. Moreover, “commode chair” was added to the list of adaptive technologies and medical aids.

The inclusion of these findings led to the final version of the RECAPDOC questionnaire. The content is presented in Table 4. In brief, the final questionnaire contains 29 items and should be used alongside other, more generic instruments to collect sociodemographic and condition-specific data or the use of drugs. The final German questionnaire and an English translation are inluded as Additional files 3 and 4.

**Table 4:** Content of the final questionnaire

Item no.	Area of assessment	Items	Response options	Time interval
1–4	Living situation	Private household; nursing or residential home; special nursing facility for people with severe DOC; assisted living community; others	Yes/no	Current situation
5–8	Utilisation of home health care and domestic services	Home health care service; paid domestic assistant; informal care by family caregivers, friends or neighbours; other forms of inpatient care e.g. day-care centre	Yes/no, Amount in days/hours/minutes per week Starting date	3 months
9–11	Social insurance benefits	Benefits of statutory long-term care insurance	Yes/no Care level Attendance allowance: € per month Hardship provision: Yes/no/unknown	Current situation
		Benefits of statutory accident insurance	Yes/no	Current situation
12	Utilisation of outpatient health care	General practitioner; internist; gynaecologist; surgeon; orthopaedist; neurologist or psychiatrist; dermatologist; ophthalmologist; urologist; dentist; psychotherapist; outpatient treatment in hospital; special outpatient clinic for people with severe DOC; others	Yes/no Number of visits	Last 3 months
13	Utilization of diagnostic measures	Electroencephalography (EEG)	Yes/no	Last 3 months
14	Utilization of adaptive technologies and medical aids	Wheelchair or multifunctional wheelchair; mobility aids e.g. hoist; standing boards; walking aids e.g. wheeled walker; bathing aids e.g. shower couch; toileting aids e.g. commode chair; hospital bed (height adjustable); positioning material e.g. bed wedges; tracheostomy equipment; suction units; ventilator; inhalation devices; feeding tube e.g. PEG-tube; feeding pump; communication devices; orthosis or splints; continence products; others	Owner: Yes/no User: Yes/no	Current situation and last 3 months
15–27	Utilization of therapies	Physical therapy; occupational therapy; speech and language therapy; rehabilitation nursing	Yes/no Starting date Amount in days/hours/minutes per week	Last 3 months
		Others	Yes/no; Amount in days/hours/minutes per week	Last 3 months
15–27	Experience of family support (see Table 1)	Information provision; competent consulting; instructions for therapeutic und nursing measures; inclusion in therapeutic process; physical and psychological relief; support for social participation (separately for physical therapy; occupational therapy; speech therapy; rehabilitation nursing)	Strongly agree/agree/disagree/ strongly disagree	Current situation
28	Multidisciplinary collaboration of health professionals	Support by a multidisciplinary team	Yes/no	Last 3 months
29		Collective treatment and care planning; collective documentation; joint team and case discussions; working across disciplines; collective conceptual orientation; no multidisciplinary team work	Strongly agree/agree/disagree/ strongly disagree/unknown	Current situation

## Discussion

To our knowledge, the RECAPDOC questionnaire is the first specific instrument to document the utilization the multitude of rehabilitation and other health care services provided for individuals with DOC in long-term care. This questionnaire is a first step to make the care of this highly vulnerable population amenable for evaluation.

When comparing the content of the initial version of the RECAPDOC questionnaire with the final tested version, minor adaptions and amendments had to be made, except in the section on special treatment concepts. Specialized treatment concepts like Facio-Oral Tract Therapy or Neurodevelopmental Treatment were identified as an important component in the rehabilitation of patients with DOC in long term both in our systematic review [20] and the expert Delphi survey. However, our pre-test revealed that documenting the utilization of those therapies is not possible by approaching the personal caregivers. Although therapeutic alliance, i.e. established and trustful communication on collaboration, task and treatment goals among patients, their families and therapists, is usually well established [29], the communication among therapists and family caregivers did not sufficiently cover specific treatment concepts in our study. To acknowledge the relevance of special treatment concepts in the treatment and management of individuals with DOC, further studies need to explore reliable ways to collect data on that from other sources, presumably from the therapists in charge.

We decided to use a 3-months retrospective approach to document health care utilization for two reasons. One important generic questionnaire for health care utilization in Germany, the FIMA [28], used the same format and a common time frame makes it easier to administer both instruments jointly. In addition, even if a shorter interval might lead to more precise information, the burden for the caregivers with filling the questionnaire should not be multiplied.

The major strength of this study is the use of an iterative and comprehensive development approach based on a systematic review of the literature, guidelines and existing questionnaires and with the involvement of all relevant user groups, including caregivers, professional nurses, therapists and physicians using different methods. Since rehabilitation care provision for individuals with DOC is considered to be multidisciplinary [22], the involvement of all relevant professionals as well as the personal caregivers aspect is indispensable. The pre-test of our questionnaire by means of cognitive interviews allowed us to evaluate comprehensibility and manageability from the perspective of the relevant users. Cognitive interviews are most valuable for questions that are complex, sensitive, and intrusive for specific groups [26, 27].

Some limitations of the study need to be acknowledged. The main challenge of the study was the recruitment of caregivers as participants for the pre-test. To revise items and eliminate problems 5 to 15 interviews are recommended [25]. Even though we had access to an established registry for DOC patients in Southern Germany [18], only four persons consented to participate, presumably due to a high personal burden [30–33]. Therefore, the results of our pre-test must be interpreted with care. However, despite the small sample size, our results were consistent among the participants and our sample was heterogeneous and covered a variety of different relevant circumstances the what is also a precondition for meaningful results from cognitive interviewing [25].

A further fact needs to be acknowledged. In 2013, a UK-based working group published a paper on challenges in developing resource use measures [34] including a best-practice guideline. Even though we were not able to include this guideline in the planning of our study because we started the project in 2013, our development process is largely in line with this paper.

Since resource utilization depends on the reimbursement principles of the respective heath care system, the use of this questionnaire is only meaningful in Germany and adaptions are necessary to use the questionnaire in other health care systems.

Further studies, e.g. addressing the economical perspective or investigating resource utilization as predictor for patient outcomes need to be done.

## Conclusions

The developed RECAPDOC questionnaire makes the situation of patients with disorders of consciousness in the long-term care setting accessible for evaluation in epidemiological studies and patient registries. The documentation of special therapeutic treatment concepts needs to be appraised in an alternative way. The questionnaire can now be used in future studies that may want to explore the association of rehabilitation interventions and patient-related outcomes.

## Additional files

Additional file 1: Questionnaires of the Delphi survey (round 1 & 2). (DOCX 15 kb)

Additional file 2: Interview guide for the expert interviews with physicians. (DOCX 13 kb)

Additional file 3: German version of the REACPDOC questionnaire. (PDF 684 kb)

Additional file 4: English translation of the RECAPDOC questionnaire. (PDF 779 kb)

## Abbreviations

DOC: Disorder of consciousness
MCS: Minimally conscious state
UWS: Unresponsive wakefulness syndrome
VS: Vegetative state

## References

[1] Bernat JL (2006). Chronic disorders of consciousness. Lancet.

[2] Hirschberg R, Giacino JT (2011). The vegetative and minimally conscious states: diagnosis, prognosis and treatment. Neurol Clin.

[3] Laureys S, Celesia GG, Cohadon F, Lavrijsen J, Leon-Carrion J, Sannita WG, Sazbon L, Schmutzhard E, von Wild KR, Zeman A (2010). Unresponsive wakefulness syndrome: a new name for the vegetative state or apallic syndrome. BMC Med.

[4] Fins JJ, Master MG, Gerber LM, Giacino JT (2007). The minimally conscious state: a diagnosis in search of an epidemiology. Arch Neurol.

[5] Tagliaferri F, Compagnone C, Korsic M, Servadei F, Kraus J (2006). A systematic review of brain injury epidemiology in Europe. Acta Neurochir.

[6] Donis J, Kraftner B (2011). The prevalence of patients in a vegetative state and minimally conscious state in nursing homes in Austria. Brain Inj.

[7] Saout V, Ombredane MP, Mouillie JM, Marteau C, Mathe JF, Richard I (2010). Patients in a permanent vegetative state or minimally conscious state in the Maine-et-Loire county of France: a cross-sectional, descriptive study. Ann Phys Rehabil Med.

[8] The Multi-Society Task Force on PVS (1994). Medical aspects of the persistent vegetative state (2). The multi-society task force on PVS. N Engl J Med.

[9] The Multi-Society Task Force on PVS (1994). Medical aspects of the persistent vegetative state (1). The multi-society task force on PVS. N Engl J Med.

[10] Katz DI, Polyak M, Coughlan D, Nichols M, Roche A (2009). Natural history of recovery from brain injury after prolonged disorders of consciousness: outcome of patients admitted to inpatient rehabilitation with 1-4 year follow-up. Prog Brain Res.

[11] Lammi MH, Smith VH, Tate RL, Taylor CM (2005). The minimally conscious state and recovery potential: a follow-up study 2 to 5 years after traumatic brain injury. Arch Phys Med Rehabil.

[12] Luaute J, Maucort-Boulch D, Tell L, Quelard F, Sarraf T, Iwaz J, Boisson D, Fischer C (2010). Long-term outcomes of chronic minimally conscious and vegetative states. Neurology.

[13] Nakase-Richardson R, Whyte J, Giacino JT, Pavawalla S, Barnett SD, Yablon SA, Sherer M, Kalmar K, Hammond FM, Greenwald B (2012). Longitudinal outcome of patients with disordered consciousness in the NIDRR TBI model systems programs. J Neurotrauma.

[14] Whyte J, Nakase-Richardson R, Hammond FM, McNamee S, Giacino JT, Kalmar K, Greenwald BD, Yablon SA, Horn LJ (2013). Functional outcomes in traumatic disorders of consciousness: 5-year outcomes from the National Institute on Disability and Rehabilitation Research traumatic brain injury model systems. Arch Phys Med Rehabil.

[15] Giacino JT, Katz DI, Whyte J (2013). Neurorehabilitation in disorders of consciousness. Semin Neurol.

[16] Bender A, Jox RJ, Grill E, Straube A, Lulé D (2015). Persistent vegetative state and minimally conscious state: a systematic review and meta-analysis of diagnostic procedures. Dtsch Arztebl Int.

[17] Klein AM, Howell K, Vogler J, Grill E, Straube A, Bender A. Rehabilitation outcome of unconscious traumatic brain injury patients. J Neurotrauma. 2013;30(17):1476–83. 10.1089/neu.2012.2735. 10.1089/neu.2012.2735 PMC3751265 23477301

[18] Grill E, Klein AM, Howell K, Arndt M, Bodrozic L, Herzog J, Jox R, Koenig E, Mansmann U, Muller F (2013). Rationale and design of the prospective German registry of outcome in patients with severe disorders of consciousness after acute brain injury. Arch Phys Med Rehabil.

[19] Howell K, Grill E, Klein AM, Straube A, Bender A (2013). Rehabilitation outcome of anoxic-ischaemic encephalopathy survivors with prolonged disorders of consciousness. Resuscitation.

[20] Klingshirn H, Grill E, Bender A, Strobl R, Mittrach R, Braitmayer K, Müller M. Quality of evidence of rehabilitation interventions in long-term care for people with severe disorders of consciousness after brain injury: a systematic review. J Rehabil Med. 2015; in press 10.2340/16501977-1983 26122074

[21] Linstone HA, Turoff M. The Delphi method: techniques and applications. Boston: Addison-Wesley Pub. Co., Advanced Book Program; 1975.

[22] Bundesarbeitsgemeinschaft für Rehabilitation. Empfehlungen zur stationären Langzeitpflege und Behandlung von Menschen mit schweren und schwersten Schädigungen des Nervensystems in der Phase F. [Recommendations for inpatient long-term care and treatment of people with severe brain damage in the phase F]. 2003. https://www.bar-frankfurt.de/fileadmin/dateiliste/publikationen/empfehlungen/downloads/Rahmenempfehlung_station%C3%A4re_Langzeitpflege.pdf. Accessed 02 May 2018.

[23] Patton MQ (1990). Qualitative evaluation and research methods.

[24] Denzin NK (1970). The research act: a theoretical introduction to sociological methods.

[25] Beatty PC, Willis GB (2007). Research synthesis: the practice of cognitive interviewing. Public Opin Q.

[26] Collins D (2003). Pretesting survey instruments: an overview of cognitive methods. Qual Life Res.

[27] Drennan J (2003). Cognitive interviewing: verbal data in the design and pretesting of questionnaires. J Adv Nurs.

[28] Seidl H, Bowles D, Bock JO, Brettschneider C, Greiner W, Konig HH, Holle R. FIMA - questionnaire for health-related resource use in an elderly population: development and pilot study. Gesundheitswesen (Bundesverband der Arzte des Offentlichen Gesundheitsdienstes (Germany)). 2014;77(1):46–52. 10.1055/s-0034-1372618 24806594

[29] Pinto RZ, Ferreira ML, Oliveira VC, Franco MR, Adams R, Maher CG, Ferreira PH (2012). Patient-centred communication is associated with positive therapeutic alliance: a systematic review. J Physiother.

[30] Doser K, Norup A (2016). Caregiver burden in Danish family members of patients with severe brain injury: the chronic phase. Brain Inj.

[31] Leonardi M, Giovannetti AM, Pagani M, Raggi A, Sattin D, National Consortium F, Disability In V, In Minimal Conscious State P (2012). Burden and needs of 487 caregivers of patients in vegetative state and in minimally conscious state: results from a national study. Brain Inj.

[32] Pagani M, Giovannetti AM, Covelli V, Sattin D, Leonardi M (2014). Caregiving for patients in vegetative and minimally conscious states: perceived burden as a mediator in caregivers’ expression of needs and symptoms of depression and anxiety. J Clin Psychol Med Settings.

[33] Pagani M, Giovannetti AM, Covelli V, Sattin D, Raggi A, Leonardi M (2014). Physical and mental health, anxiety and depressive symptoms in caregivers of patients in vegetative state and minimally conscious state. Clin Psychol Psychother.

[34] Thorn JC, Coast J, Cohen D, Hollingworth W, Knapp M, Noble SM, Ridyard C, Wordsworth S, Hughes D (2013). Resource-use measurement based on patient recall: issues and challenges for economic evaluation. Appl Health Econ Health Policy.

