# Supplementary material for: RECAPDOC - a questionnaire for the documentation of rehabilitation care utilization in individuals with disorders of consciousness in long-term care in Germany: development and pretesting
Source: BMC Health Serv Res. 2018 May 4;18:329. doi: 10.1186/s12913-018-3153-3 (PMC5936017; doi:10.1186/s12913-018-3153-3)
Supplement: Supplementary file 1 — Questionnaires of the Delphi survey (round 1 & 2). (DOCX 15 kb) [file 12913_2018_3153_MOESM1_ESM.docx]

# Questions of the Delphi survey (round 1)

1. How old are you? [years]
2. Please indicate your gender. [Response options: male, female]
3. What is your profession? [Response options: Nursing, physical therapy, occupational therapy, speech and language therapy, other]
4. How long have you been working in rehabilitation of individuals with disorders of consciousness? [years]
5. How long have you been working rehabilitation of individuals with disorders of consciousness in long-term care? [years]
6. What is the main setting that you are working in? [Response options: community nursing, outpatient rehabilitation, outpatient social care service, day care, specialized nursing home/care facility for DOC, nursing home]
7. Which relevant trainings for neurological rehabilitation did you complete?
8. Which special treatment concepts for individuals with DOC do you apply?
9. Which medical aids do you use or prescibe?
10. What kind of support do you offer to lay/ family carers?
11. How do you facilitate social participation of individuals with DOC?
12. With which professions do you collaborate in the care of individuals with DOC?
13. What are the main factors that facilitate interprofessional collaboration in the care of individuals with DOC?

# Questions of the Delphi survey (round 2)

1. How old are you? [years]
2. Please indicate your gender. [Response options: male, female]
3. What is your profession? [Response options: Nursing, physical therapy, occupational therapy, speech and language therapy, other]
4. How long have you been working in rehabilitation of individuals with disorders of consciousness? [years]
5. How long have you been working rehabilitation of individuals with disorders of consciousness in long-term care? [years]
6. What is the main setting that you are working in? [Response options: community nursing, outpatient rehabilitation, outpatient social care service, day care, specialized nursing home/care facility for DOC, nursing home]
7. How do you rate your personal competency regarding the following special treatment concepts? [response options: Expert knowledge, advanced knowledge, basic knowledge, none]
   1. Bobath/ neuro-developmental treatment
   2. Facio-oral-tract therapy
   3. Basal stimulation
   4. Affolter model
   5. Kinaestetics
   6. Positioning in neutral
   7. Music therapy
   8. Neurodynamics
   9. Dysphagia therapy
   10. VeReGo concept
8. How often are the following special treatment concepts been used in your working area? [response options: always, often, sometimes, never]
   1. Bobath/ neuro-developmental treatment
   2. Facio-oral-tract therapy
   3. Basal stimulation
   4. Affolter model
   5. Kinaestetics
   6. Positioning in neutral
   7. Music therapy
   8. Neurodynamics
   9. Dysphagia therapy
   10. VeReGo concept
9. Are there any other special treatment concepts that were “always” or “often” used in your working area?
10. How do you personally rate the relevance of the following special treatment concepts for individuals with DOC in long-term care? [response options: very relevant, relevant, less relevant, not relevant at all, can’t judge]
    1. Bobath/ neuro-developmental treatment
    2. Facio-oral-tract therapy
    3. Basal stimulation
    4. Affolter model
    5. Kinaestetics
    6. Positioning in neutral
    7. Music therapy
    8. Neurodynamics
    9. Dysphagia therapy
    10. VeReGo concept
11. Are there any other special treatment concepts that you consider as “very relevant” or “relevant” in your working area?
12. How do you personally rate the relevance of the following medial aids for the care of individuals with DOC in long-term care? [response options: very relevant, relevant, less relevant, not relevant at all, can’t judge]
    1. Wheel chairs
    2. positioning materials
    3. suction units
    4. hospital beds
    5. tracheostomy equipment
    6. bathroom hoists and seats
    7. standing boards
    8. communication devices
    9. mobility devices
    10. orthotics/ splints
    11. walking aids
    12. feeding devices
13. Are there any other medical aids that you consider as “very relevant” or “relevant” in your working area?
14. How do you rate the following measures to support family/ lay carers? [response options: very important, important, less important, not important at all, can’t judge]
    1. Comprehensive medical information
    2. Consultation regarding rehabilitation measures, medical aids, alternative living arrangements, self-help groups
    3. Consultation and training regarding patient handling and use of medical aids
    4. Close involvement into the caring and rehabilitation process
    5. Relief from physical and psychological strain due to caring for a family member
    6. Support regarding facilitation of social participation of the individual with DOC
15. How do you rate the relevance of the following rehabilitation goals for individuals with DOC in long-term care? [response options: very important, important, less important, not important at all, can’t judge]
    1. Sensual perception
    2. Communication
    3. Mobility
    4. Self-care
    5. Social participation
16. How do you rate the relevance of the following measures for interdisciplinary collaboration in long-term care for individuals with DOC? [response options: very important, important, less important, not important at all, can’t judge]
    1. Close involvement of the family
    2. Joint care planning
    3. Team meetings and case conferences
    4. Joint therapy
    5. Joint documentation
    6. Interdisciplinary training on a regular basis
    7. Common standards of care
    8. Supportive environment (available time, financial support, built environment)
    9. Positive working atmosphere
