# Supplementary material for: RECAPDOC - a questionnaire for the documentation of rehabilitation care utilization in individuals with disorders of consciousness in long-term care in Germany: development and pretesting
Source: BMC Health Serv Res. 2018 May 4;18:329. doi: 10.1186/s12913-018-3153-3 (PMC5936017; doi:10.1186/s12913-018-3153-3)
Supplement: Supplementary file 2 — Interview guide for the expert interviews with physicians. (DOCX 13 kb) [file 12913_2018_3153_MOESM2_ESM.docx]

# Interview guide for the expert interviews with physicians

Medical specialities

1. Are there any medical specialists relevant for the long-term care of individuals with DOC that are not listed in our questionnaire? If so, which ones?
2. Are there any medical specialists listed that are not relevant for the long-term care of individuals with DOC? If so, which ones?
3. Do you have further remarks regarding medical specialists?

Medical aids

1. Are there any medical aids relevant for the long-term care of individuals with DOC that are not listed in our questionnaire? If so, which ones?
2. Are there any medical aids listed that are not relevant for the long-term care of individuals with DOC? If so, which ones?
3. Do you have further remarks regarding medical aids?

Special treatment concepts

1. Are there any special treatment concepts relevant for the long-term care of individuals with DOC that are not listed in our questionnaire? If so, which ones?
2. Are there any special treatment concepts aids listed that are not relevant for the long-term care of individuals with DOC? If so, which ones?
3. Do you have further remarks regarding special treatment concepts?

Additional therapy

1. Are there any therapies relevant for the long-term care of individuals with DOC that are not listed in our questionnaire? If so, which ones?
