# Supplementary material for: RECAPDOC - a questionnaire for the documentation of rehabilitation care utilization in individuals with disorders of consciousness in long-term care in Germany: development and pretesting
Source: BMC Health Serv Res. 2018 May 4;18:329. doi: 10.1186/s12913-018-3153-3 (PMC5936017; doi:10.1186/s12913-018-3153-3)
Supplement: Supplementary file 3 — German version of the REACPDOC questionnaire. (PDF 684 kb) [file 12913_2018_3153_MOESM3_ESM.pdf]

LUDWIG-
MAXIMILIANS-
UNIVERSITÄT
MÜNCHEN

INSTITUT FÜR MEDIZINISCHE
INFORMATIONSVERARBEITUNG, BIOMETRIE
UND EPIDEMIOLOGIE

Fragebogen zur Inanspruchnahme rehabilitativer Leistungen in der Langzeitversorgung von Menschen mit schweren Bewusstseinsstörungen

Hinweise zum Ausfüllen des Fragebogens

© Institut für Medizinische Informationsverarbeitung, Biometrie und Epidemiologie (IBE) der Ludwig-Maximilians-Universität München

Kontakt: martin.mueller@fh-rosenheim.de

Gefördert durch die Deutsche Forschungsgemeinschaft

Bitte lassen Sie sich von dem Umfang des Fragebogens nicht abschrecken!
Sie werden insgesamt etwa 20 Minuten für Ihre Antworten benötigen.

So wird's gemacht!

Bitte füllen Sie den Fragebogen aus, indem Sie

- In den Kästchen ein Kreuz machen

  Beispiel: Nein ☒ Ja ☐

- In den Feldern eintragen

  Beispiel: durchschnittlich | _ | 1 | Stunden und | 3 | 0 | Minuten

- Angaben in Druckbuchstaben auf der vorgegebenen Linie machen

  Beispiel: Sonstige: Betreutes Wohnen

Im Fragebogen werden verschiedene Zeiträume abgefragt (3 Monate und 12 Monate). Bitte achten Sie bei den jeweiligen Fragen, auf welchen Zeitraum sich diese beziehen und nehmen Sie bitte einen **Kalender zu Hilfe**, um die zurückliegenden Zeiten zu bestimmen.

Gehen Sie bitte der Reihe nach vor und beantworten Sie wenn möglich alle Fragen.

Vielen Dank!

1) Welche Hauptdiagnose liegt der Bewusstseinsstörung des Betroffenen zugrunde?

Bitte Entsprechendes ankreuzen.

Schädel-Hirn-Trauma	☐
Schlaganfall	☐
Hirnblutung (intrazerebral oder subarachnoidal)	☐
Sauerstoffmangel im Gehirn	☐
Sonstige: ______________________________	☐

2) Wann war das Datum der Schädigung des Betroffenen?

☐☐ ☐☐ ☐☐☐☐

Tag Monat Jahr

3) Wann ist der Betroffene aus der stationären Rehabilitation entlassen worden?

☐☐ ☐☐ ☐☐☐☐

Tag Monat Jahr

4) Wo wohnt der Betroffene **zurzeit**?

Bitte Entsprechendes ankreuzen.

Privater Haushalt	☐
Senioren-/Pflegeheim	☐
Fachpflegeeinrichtung für Menschen mit schweren Bewusstseinsstörungen	☐
Betreute Wohngemeinschaft	☐
Sonstige: ______________________________	☐

5) Hat der Betroffene in den **letzten 3 Monaten** einen ambulanten Pflegedienst in Anspruch genommen?

Bitte Entsprechendes ankreuzen und ausfüllen.

- ☐ Nein
- ☐ Ja →

Wenn ja, seit wann nimmt der Betroffene den ambulanten Pflegedienst in Anspruch?

|_|_| |_|_|_|_|
Monat Jahr

An wie vielen Tagen pro Woche nimmt der Betroffene den ambulanten Pflegedienst in Anspruch?

an |_| Tagen pro Woche

An diesen Tagen, wie lange war der ambulante Pflegedienst durchschnittlich bei dem Betroffenen zu Hause?

durchschnittlich |_|_| Stunden und |_|_| Minuten

6) Hat der Betroffene in den **letzten 3 Monaten** eine bezahlte Haushalts- oder Pflegehilfe in Anspruch genommen?

Bitte Entsprechendes ankreuzen und ausfüllen.

- ☐ Nein
- ☐ Ja →

Wenn ja, seit wann nimmt der Betroffene die bezahlte Haushalts- oder Pflegehilfe in Anspruch?

|_|_| |_|_|_|_|
Monat Jahr

An wie vielen Tagen pro Woche nimmt der Betroffene die bezahlte Haushalts- oder Pflegehilfe in Anspruch?

an |_| Tagen pro Woche

An diesen Tagen, wie lange war die bezahlte Haushalts- oder Pflegehilfe durchschnittlich bei dem Betroffenen zu Hause?

durchschnittlich |_|_| Stunden und |_|_| Minuten

7) Hat der Betroffene in den **letzten 3 Monaten** Hilfen von Familienmitgliedern, Freunden, Bekannten oder Nachbarn in Anspruch genommen?

Beispiele für diese Hilfen sind: Hilfen im Haushalt, bei der Körperpflege, beim An- und Auskleiden, bei der Medikamenteneinnahme, beim Einkaufen, bei Fahrdiensten

Bitte Entsprechendes ankreuzen und ausfüllen.

☐ Nein

☐ Ja →

Wenn ja, seit wann nimmt der Betroffene diese Hilfen in Anspruch?

|_|_| |_|_|_|_|
Monat Jahr

An wie vielen Tagen pro Woche nimmt der Betroffene diese Hilfen in Anspruch?

an |_| Tagen pro Woche

An diesen Tagen, wie lange wurde dem Betroffenen durchschnittlich geholfen?

durchschnittlich |_|_| Stunden und |_|_| Minuten

8) Hatte der Betroffene in den **letzten 3 Monaten** einen Aufenthalt in einer teilstationären Pflegeeinrichtung (Tagespflege)?

Bitte Entsprechendes ankreuzen und ausfüllen.

☐ Nein

☐ Ja →

Wenn ja, seit wann besucht der Betroffene diese teilstationäre Pflegeeinrichtung (Tagespflege)?

|_|_| |_|_|_|_|
Monat Jahr

An wie vielen Tagen pro Woche besucht der Betroffene die teilstationäre Pflegeeinrichtung (Tagespflege)?

an |_| Tagen pro Woche

An diesen Tagen, wie lange besucht der Betroffene durchschnittlich die teilstationäre Pflegeeinrichtung (Tagespflege)?

durchschnittlich |_|_| Stunden und |_|_| Minuten

9) Erhält der Betroffene Leistungen aus der gesetzlichen Pflegeversicherung?

Bitte Entsprechendes ankreuzen und ausfüllen.

☐ Nein

☐ Ja →

Wenn ja, welche Pflegestufe hat der Betroffene?

Pflegestufe ☐

Falls Ihnen die Pflegestufe des Betroffenen nicht bekannt ist: Wie hoch ist das monatliche Pflegegeld?

☐☐☐☐ Euro

Falls der Betroffene in Pflegestufe 3 eingruppiert ist: Wird die **Härtefallregelung** (höhere Sachleistungen) in Anspruch genommen?

Härtefallregelung: Nein ☐ Ja ☐ Weiß nicht ☐

10) Erhält der Betroffene Leistungen aus der gesetzlichen Unfallversicherung?

Bitte Entsprechendes ankreuzen.

☐ Nein ☐ Ja

11) Wie ist der Betroffene krankenversichert?

Bitte Entsprechendes ankreuzen.

Gesetzlich Versichert	☐
Über Beihilfe und privat versichert (Beamtenstatus)	☐
Privat versichert (nur Vollversicherung, keine Zusatzversicherung)	☐
Keine Krankenversicherung	☐

12) Hat der Betroffene in den **letzten 3 Monaten** einen der folgenden Ärzte aufgesucht?
Gemeint ist jeder Kontakt mit der Arztpraxis, auch wenn der Arzt selbst nicht gesprochen wurde (zum Beispiel Rezept abholen, Blutabnahme). Hausbesuche zählen auch dazu.
Bitte kreuzen Sie zunächst an, ob der Betroffene den jeweiligen Arzt aufgesucht hat (Ja oder Nein).
Wenn ja, dann geben Sie bitte weiterhin an, wie oft der Betroffene diesen Arzt aufgesucht hat.

Arzt	Nein	Ja	Wie oft?
Allgemeinmediziner, Hausarzt oder hausärztlicher Internist	☐	☐	☐☐
Fachärztlicher Internist (z.B. Kardiologe, Gastroenterologe, Nephrologe, Diabetologe, Pulmologe, usw.)	☐	☐	☐☐
Gynäkologe	☐	☐	☐☐
Chirurg	☐	☐	☐☐
Orthopäde	☐	☐	☐☐
Neurologe / Psychiater	☐	☐	☐☐
Hautarzt	☐	☐	☐☐
Augenarzt	☐	☐	☐☐
Urologe	☐	☐	☐☐
Zahnarzt	☐	☐	☐☐
Psychotherapeut	☐	☐	☐☐
Ambulante Behandlung im Krankenhaus (z.B. Sprechstunde, Notfallversorgung, Vor- und Nachsorge bei Operationen)	☐	☐	☐☐
An eine Klinik angebundene Spezialambulanz für Menschen mit schweren Bewusstseinsstörungen	☐	☐	☐☐
Sonstige: ________________	☐	☐	☐☐

13) Wurde bei dem Betroffenen in den **letzten 3 Monaten** eine Hirnstrommessung (EEG, Elektroenzephalografie) durchgeführt?

Bitte Entsprechendes ankreuzen.

☐ Nein ☐ Ja

14) Besitzt der Betroffene eines oder mehrere der folgenden **Hilfsmittel**? Dies schließt gekaufte oder geliehene Hilfsmittel ein.
Bitte kreuzen Sie zunächst jeweils an, ob der Betroffene das Hilfsmittel besitzt.
Wenn ja, geben Sie bitte weiterhin an, ob der Betroffene das Hilfsmittel in den **letzten 3 Monaten** verwendet hat.

Hilfsmittel	Nein	Ja, besitzt dieses	Ja, in den letzten 3 Monaten verwendet
Rollstuhl, Multifunktions- oder Pflegerollstuhl	☐	☐	☐
Transferhilfen z.B. Lifter	☐	☐	☐
Stehhilfen z.B. Stehständer	☐	☐	☐
Gehhilfen z.B. Rollator	☐	☐	☐
Badehilfen z.B. Dusch- oder Badewannenliege	☐	☐	☐
WC-Hilfen z.B. WC-Stuhl	☐	☐	☐
Behindertengerechtes Bett (höhenverstellbar)	☐	☐	☐
Lagerungshilfen z.B. Kissen, Keile	☐	☐	☐
Hilfsmittel bei Tracheostoma	☐	☐	☐
Sekret-Absauggerät	☐	☐	☐
Beatmungsgerät	☐	☐	☐
Inhalationsgerät	☐	☐	☐
Ernährungssonde z.B. PEG-Sonde	☐	☐	☐
Ernährungspumpe z.B. PEG-Pumpe	☐	☐	☐
Kommunikationshilfen z.B. technisch, symbolisch	☐	☐	☐
Orthesen oder Schienen	☐	☐	☐
Hilfsmittel zur Inkontinenzversorgung	☐	☐	☐
Sonstige: ______________________	☐	☐	☐
Sonstige: ______________________	☐	☐	☐

15) Hat der Betroffene in den **letzten 3 Monaten Physiotherapie** in Anspruch genommen? Hausbesuche zählen auch dazu.

Bitte Entsprechendes ankreuzen.

- ☐ Nein ⟶ dann weiter mit Frage **18)**
- ☐ Ja ⟶ dann weiter mit Frage **16)**

16) Wenn der Betroffene in den **letzten 3 Monaten Physiotherapie** in Anspruch genommen hat, beantworten Sie bitte folgende Fragen zum zeitlichen Umfang der Behandlung?

Bitte Entsprechendes ankreuzen und ausfüllen.

Seit wann nimmt der Betroffene Physiotherapie in Anspruch?

|_|_| |_|_|_|_|
Monat Jahr

An wie vielen Tagen pro Woche nimmt der Betroffene Physiotherapie in Anspruch?

an |_| Tagen pro Woche

An diesen Tagen, wie lange dauert die Physiotherapie durchschnittlich?

durchschnittlich |_|_| Stunden und |_|_| Minuten

Wird die Physiotherapie langfristig verordnet d.h. außerhalb des Regelfalles und **ohne** mindestens dreimonatige Therapiepausen?

Nein ☐ Ja ☐

17) **Als Angehörige**, wie beurteilen Sie folgende Aussagen bezüglich der **Physiotherapie** des Betroffenen?

Bitte Entsprechendes ankreuzen.

**Durch die Physiotherapie…**	trifft zu	trifft eher zu	trifft eher nicht zu	trifft nicht zu
werde ich ausreichend informiert.	☐	☐	☐	☐
werde ich kompetent beraten.	☐	☐	☐	☐
erhalte ich eine Anleitung zur Umsetzung therapeutischer oder pflegerischer Maßnahmen.	☐	☐	☐	☐
werde ich in den Therapieprozess miteingebunden.	☐	☐	☐	☐
werde ich psychisch und physisch entlastet.	☐	☐	☐	☐
wird mir und dem Betroffenen eine Teilhabe am sozialen Leben ermöglicht.	☐	☐	☐	☐

18) Hat der Betroffene in den **letzten 3 Monaten Ergotherapie** in Anspruch genommen? Hausbesuche zählen auch dazu.

Bitte Entsprechendes ankreuzen.

☐ Nein → dann weiter mit Frage **21)**

☐ Ja → dann weiter mit Frage 19**)**

19) Wenn der Betroffene in den **letzten 3 Monaten Ergotherapie** in Anspruch genommen hat, beantworten Sie bitte folgende Fragen zum zeitlichen Umfang der Behandlung?

Bitte Entsprechendes ankreuzen und ausfüllen.

Seit wann nimmt der Betroffene Ergotherapie in Anspruch?

|_|_| |_|_|_|_|
Monat Jahr

An wie vielen Tagen pro Woche nimmt der Betroffene Ergotherapie in Anspruch?

an |_| Tagen pro Woche

An diesen Tagen, wie lange dauert die Ergotherapie durchschnittlich?

durchschnittlich |_|_| Stunden und |_|_| Minuten

Wird die Ergotherapie langfristig verordnet d.h. außerhalb des Regelfalles und **ohne** mindestens dreimonatige Therapiepausen?

Nein ☐ Ja ☐

20) Als Angehörige, wie beurteilen Sie folgende Aussagen bezüglich der **Ergotherapie** des Betroffenen?

Bitte Entsprechendes ankreuzen.

**Durch die Ergotherapie…**	trifft zu	trifft eher zu	trifft eher nicht zu	trifft nicht zu
werde ich ausreichend informiert.	☐	☐	☐	☐
werde ich kompetent beraten.	☐	☐	☐	☐
erhalte ich eine Anleitung zur Umsetzung therapeutischer oder pflegerischer Maßnahmen.	☐	☐	☐	☐
werde ich in den Therapieprozess miteingebunden.	☐	☐	☐	☐
werde ich psychisch und physisch entlastet.	☐	☐	☐	☐
wird mir und dem Betroffenen eine Teilhabe am sozialen Leben ermöglicht.	☐	☐	☐	☐

21) Hat der Betroffene in den **letzten 3 Monaten Stimm-, Sprech- und Sprachtherapie** in Anspruch genommen? Hausbesuche zählen auch dazu.

Bitte Entsprechendes ankreuzen.

☐ Nein ⟶ dann weiter mit Frage **24)**

☐ Ja ⟶ dann weiter mit Frage **22)**

22) Wenn der Betroffene in den **letzten 3 Monaten Stimm-, Sprech- und Sprachtherapie** in Anspruch genommen hat, beantworten Sie bitte folgende Fragen zum zeitlichen Umfang der Behandlung?

Bitte Entsprechendes ankreuzen und ausfüllen.

Seit wann nimmt der Betroffene Stimm-, Sprech- und Sprachtherapie in Anspruch?

|_|_| |_|_|_|_|
Monat Jahr

An wie vielen Tagen pro Woche nimmt der Betroffene Stimm-, Sprech- und Sprachtherapie in Anspruch?

an |_| Tagen pro Woche

An diesen Tagen, wie lange dauert die Stimm-, Sprech- und Sprachtherapie durchschnittlich?

durchschnittlich |_|_| Stunden und |_|_| Minuten

Wird die Stimm-, Sprech- und Sprachtherapie langfristig verordnet d.h. außerhalb des Regelfalles und **ohne** mindestens dreimonatige Therapiepausen?

Nein ☐ Ja ☐

23) Als Angehörige, wie beurteilen Sie folgende Aussagen bezüglich der **Stimm-, Sprech- und Sprachtherapie** des Betroffenen?

Bitte Entsprechendes ankreuzen.

**Durch die Stimm-, Sprech- und Sprachtherapie…**	trifft zu	trifft eher zu	trifft eher nicht zu	trifft nicht zu
werde ich ausreichend informiert.	☐	☐	☐	☐
werde ich kompetent beraten.	☐	☐	☐	☐
erhalte ich eine Anleitung zur Umsetzung therapeutischer oder pflegerischer Maßnahmen.	☐	☐	☐	☐
werde ich in den Therapieprozess miteingebunden.	☐	☐	☐	☐
werde ich psychisch und physisch entlastet.	☐	☐	☐	☐
wird mir und dem Betroffenen eine Teilhabe am sozialen Leben ermöglicht.	☐	☐	☐	☐

24) Hat der Betroffene in den **letzten 3 Monaten professionelle Pflege** (gemeint ist damit eine Begleitung durch ausgebildete Fachpflegekräfte) in Anspruch genommen? Hausbesuche zählen auch dazu.

Bitte Entsprechendes ankreuzen.

- ☐ Nein ⟶ dann weiter mit Frage **27)**
- ☐ Ja ⟶ dann weiter mit Frage **25)**

25) Wenn der Betroffene in den **letzten 3 Monaten professionelle Pflege** in Anspruch genommen hat, beantworten Sie bitte folgende Fragen zum zeitlichen Umfang der Behandlung?

Bitte Entsprechendes ankreuzen und ausfüllen.

Seit wann nimmt der Betroffene professionelle Pflege in Anspruch?

|_|_| |_|_|_|_|
Monat Jahr

An wie vielen Tagen pro Woche nimmt der Betroffene professionelle Pflege in Anspruch?

an |_| Tagen pro Woche

An diesen Tagen, wie lange dauert die professionelle Pflege durchschnittlich?

durchschnittlich |_|_| Stunden und |_|_| Minuten

Findet die professionelle Pflege langfristig d.h. **ohne** Versorgungspausen statt?

Nein ☐ Ja ☐

26) **Als Angehörige**, wie beurteilen Sie folgende Aussagen bezüglich der **professionelle Pflege** des Betroffenen?

Bitte Entsprechendes ankreuzen.

**Durch die professionelle Pflege…**	trifft zu	trifft eher zu	trifft eher nicht zu	trifft nicht zu
werde ich ausreichend informiert.	☐	☐	☐	☐
werde ich kompetent beraten.	☐	☐	☐	☐
erhalte ich eine Anleitung zur Umsetzung therapeutischer oder pflegerischer Maßnahmen.	☐	☐	☐	☐
werde ich in den Therapieprozess miteingebunden.	☐	☐	☐	☐
werde ich psychisch und physisch entlastet.	☐	☐	☐	☐
wird mir und dem Betroffenen eine Teilhabe am sozialen Leben ermöglicht.	☐	☐	☐	☐

27) Hat der Betroffene in den **letzten 3 Monaten sonstige therapeutische Angebote** (z.B. Musiktherapie, tiergestützte Therapie) in Anspruch genommen? Hausbesuche zählen auch dazu.

Bitte geben tragen Sie die sonstigen therapeutischen Angebote die der Betroffene in Anspruch genommen hat zunächst ein, und geben Sie weiterhin an, wie oft der Betroffene diese Angebote wahrgenommen hat.

Bitte Entsprechendes ankreuzen und ausfüllen.

☐ Nein

☐ Ja →

Sonstiges therapeutisches Angebot	Wie oft?
______________________	└┴┘
______________________	└┴┘
______________________	└┴┘

28) Hat der Betroffene in den **letzten 3 Monaten** Leistungen eines **multidisziplinären Reha-Teams** in Anspruch genommen? Hausbesuche zählen auch dazu.

Gemeint ist die Inanspruchnahme verschiedener rehabilitativer Leistungen (z.B. Physio- und Ergotherapie), auch wenn die Pflegekräfte und Therapeuten aus verschiedenen Einrichtung stammen oder externe Pflegekräfte und Therapeuten zur Verstärkung des Reha-Teams hinzugezogen werden.

Bitte Entsprechendes ankreuzen.

☐ Nein ⟶ dann weiter mit Frage **34)**

☐ Ja ⟶ dann weiter mit Frage **33)**

29) **Als Angehörige**, wie beurteilen Sie folgende Aussagen bezüglich der **Zusammenarbeit innerhalb des Reha-Teams**?

Bitte Entsprechendes ankreuzen.

**Das Reha-Team…**	trifft zu	trifft eher zu	trifft eher nicht zu	trifft nicht zu	weiß nicht
verfolgt eine gemeinsame Pflege- und Therapieplanung.	☐	☐	☐	☐	☐
verwendet eine gemeinsame Dokumentation.	☐	☐	☐	☐	☐
gestaltet gemeinsame Team- und Fallbesprechungen.	☐	☐	☐	☐	☐
arbeitet fachübergreifend mit dem Betroffenen (z.B. Behandlung mit zwei Therapeuten unterschiedlicher Disziplin)	☐	☐	☐	☐	☐
arbeitet nach einer gemeinsamen konzeptionellen Ausrichtung.	☐	☐	☐	☐	☐
Eine Zusammenarbeit innerhalb des Reha-Teams ist für mich **nicht** ersichtlich.	☐	☐	☐	☐	☐

30) Müssen für die Versorgung des Betroffenen eigene Finanzmittel des Haushalts aufgewendet werden? Zuzahlungen der gesetzlichen Krankenversicherung zählen auch dazu.

Bitte Entsprechendes ankreuzen und ausfüllen.

☐ Nein

☐ Ja → Wenn ja, wie viel Euro pro Monat müssen durchschnittlich aufgewendet werden?

durchschnittlich |_|_|_|_| Euro pro Monat

31) Wann ist der Betroffene geboren?

|_|_| |_|_| |_|_|_|_|

Tag Monat Jahr

32) Welches Geschlecht hat der Betroffene?

☐ weiblich

☐ männlich

33) Welchen Familienstand hat der Betroffene?

verheiratet ☐

ledig ☐

geschieden ☐

verwitwet ☐

34) Welches ist der **höchste** Schulabschluss des Betroffenen?

- Hauptschule/Volksschule ☐
- Mittlere Reife/Realschule ☐
- Abitur/Fachabitur/Fachhochschulreife ☐
- kein Abschluss ☐
- Sonstiger Abschluss ____________________

35) Welches ist der **höchste** berufsbildende Schulabschluss des Betroffenen?

- Berufsschule (Lehre) ☐
- Fachschule/Techniker-/Meisterschule ☐
- Ingenieur-Schule/Polytechnikum ☐
- Hochschule/Fachhochschule/Universität ☐
- kein Abschluss ☐
- Sonstiger Abschluss ____________________

36) Bitte schätzen Sie ein, wie schwierig es für Sie war, den Fragebogen auszufüllen.

- sehr einfach ☐
- einfach ☐
- schwierig ☐
- sehr schwierig ☐
- ohne Hilfe unmöglich ☐

37) Wie viel Zeit haben Sie ungefähr benötigt, um den Fragebogen auszufüllen?

☐☐ Minuten

38) Wenn Sie möchten, können Sie hier noch Kommentare zum Inhalt, Schwierigkeitsgrad, zur Übersichtlichkeit des Fragebogens oder Ähnliches einfügen.

Sollten Sie Anmerkungen zu einzelnen Fragen des Fragebogens haben, geben Sie bitte die Nummer der Frage auf die sich Ihre Anmerkung bezieht mit an.

Bitte tragen Sie hier Ihre Kommentare ein.

39) Bitte geben Sie das heutige Datum an.

Heutiges Datum ☐☐ ☐☐ ☐☐☐☐

Tag Monat Jahr

Bitte prüfen Sie, ob Sie keine Frage übersehen haben.

Vielen Dank für Ihre Unterstützung!
