# Supplementary material for: RECAPDOC - a questionnaire for the documentation of rehabilitation care utilization in individuals with disorders of consciousness in long-term care in Germany: development and pretesting
Source: BMC Health Serv Res. 2018 May 4;18:329. doi: 10.1186/s12913-018-3153-3 (PMC5936017; doi:10.1186/s12913-018-3153-3)
Supplement: Supplementary file 4 — English translation of the RECAPDOC questionnaire. (PDF 779 kb) [file 12913_2018_3153_MOESM4_ESM.pdf]

LMU

LUDWIG-MAXIMILIANS-UNIVERSITÄT MÜNCHEN

INSTITUT FÜR MEDIZINISCHE INFORMATIONSVERARBEITUNG, BIOMETRIE UND EPIDEMIOLOGIE

RECAPDOC
Questionnaire for the documentation of rehabilitation care utilization in individuals with disorders of consciousness in long-term care

© Institute for Medical Information Processing, Biometrics and Epidemiology (IBE)
Ludwig-Maximilians-Universität München, Germany

Contact: martin.mueller@fh-rosenheim.de

Funded by Deutsche Forschungsgemeinschaft

DFG Deutsche Forschungsgemeinschaft

Instructions for completing the questionnaire

Please do not let the length of the questionnaire discourage you!
You will need about 20 minutes to complete the questionnaire.

Here is how to do it!

Please complete the questionnaire by

- writing an X in the desired answer choice

  Example: No ☒ Yes ☐

- Enter in the fields

  Example: on average | |1| Hour und |3|0| Minutes

- Enter answer in your own writing on the given line

  Example: Other: Assisted Living

The questionnaire asks about different time periods (3 months and 12 months). Please pay attention and read carefully which respective period of time is being referred to, and please use a calendar to determine the time period in question.

Please proceed in order and answer all questions if possible.

Thank you very much!

1) What is the main diagnosis underlying the patient's consciousness disorder?
Please cross the appropriate box.

- Traumatic Brain Injury ☐
- Stroke ☐
- Cerebral haemorrhage (intracerebral or subaranchnoidal) ☐
- Oxygen deficiency in the brain ☐
- Other: ____________________ ☐

2) When was the date of injury of the person concerned?

☐☐ ☐☐ ☐☐☐☐
Day Month Year

3) When was the patient discharged from inpatient rehabilitation?

☐☐ ☐☐ ☐☐☐☐
Day Month Year

4) Where does the person concerned **currently** live?

Please cross the appropriate box.

- **Private household** ☐
- **Senior/Nursing home** ☐
- **Special care facility for people with severe consciousness disorders** ☐
- **Assisted living residence** ☐
- **Other:** ____________________ ☐

5) Has the person concerned used a home care service in the **last 3 months**?

Please cross and fill out the appropriate form.

☐ No

☐ Yes → If yes, since when does the person concerned use the home care service?

|_|_| |_|_|_|_|
Month Year

On how many days a week did the person concerned use the home care service?

on |_| Days per week

On those days, how long was the home care service on average at the person of concern's home?

On average |_|_| Hours and |_|_| Minutes

6) Has the person concerned used paid household or extended care in the **last 3 months**?

Please cross and fill out the appropriate form.

☐ No

☐ Yes → If yes, since when does the person concerned use the paid household or extended care?

|_|_| |_|_|_|_|
Month Year

On how many days a week did the person concerned use the paid household or extended care?

on |_| Days per week

On those days, how long was the paid household or extended care at the person of concern's home?

On average |_|_| Hours and |_|_| Minutes

7) Has the person concerned used help from family members, friends, acquaintances or neighbors in the **last 3 months**?

Examples of help are: household help, personal hygiene, dressing and undressing, taking medication, shopping, and driving

Please cross and fill out the appropriate form.

☐ No

☐ Yes →

If yes, since when does the person concerned use this help?

Month __ __ Year __ __ __ __

On how many days a week did the person concerned use this help?

on __ Days per week

On those days, how long was the person on average helped?

on average __ __ Hours and __ __ Minutes

8) Did the person concerned stay in a semi-residential care facility (day care) in the **last 3 months**?

Please cross and fill out the appropriate form

☐ No

☐ Yes →

If so, since when does the person concerned visit this semi-residential care facility (day care)?

Month __ __ Year __ __ __ __

On how many days a week did the person concerned visit the semi-residential care facility (day care)?

on __ Days per week

On those days, how long did the person concerned visit the semi-residential care facility (day care) on average?

on average __ __ Hours and __ __ Minutes

9) Does the person concerned receive benefits from the statutory long-term care insurance?

Please cross and fill out the appropriate form.

- ☐ Nein
- ☐ Ja →

If yes, which level of care does the person concerned have?

Level of care ☐

If you do not know the care level of the person concerned: What is the monthly care allowance?

_ _ _ _ Euro

If the person concerned is in care level 3: Is the hardship clause (higher allowance in kind) used?

Hardship provision: No ☐ Yes ☐ Do not know ☐

10) Does the person concerned receive benefits from the statutory accident insurance?

Please cross the appropriate box.

☐ No ☐ Yes

11) How is the person concerned covered by health insurance?

Please cross the appropriate box.

Statutory health insurance	☐
Through assistance and privately insured (civil servant status)	☐
Private insurance (only full insurance, no additional insurance)	☐
No health insurance	☐

12) Has the person concerned visited one of the following doctors in the **last 3 months**?
This refers to any contact with the doctor's office, even if the doctor himself was not spoken to (for example picking up a prescription, taking blood). Home visits are also included.

Please cross first whether the person concerned has visited the respective doctor (yes or no).
If yes, please continue to state how often the person has visited this doctor.

Doctor	No	Yes	How often?
General practitioner, family doctor, or primary care internist	☐	☐	☐☐
Specialist internist (e.g., cardiologist, gastroenterologist, nephrologist, diabetologist, pulmonologist, etc.)	☐	☐	☐☐
Gynecologist	☐	☐	☐☐
Surgeon	☐	☐	☐☐
Orthopedist	☐	☐	☐☐
Neurologist / Psychiatrist	☐	☐	☐☐
Dermatologist	☐	☐	☐☐
Ophthalmologist	☐	☐	☐☐
Urologist	☐	☐	☐☐
Dentist	☐	☐	☐☐
Psychotherapist	☐	☐	☐☐
Outpatient treatment in the hospital (for example: consultation, emergency care, before- and after surgery)	☐	☐	☐☐
Special outpatient clinic for people with severe consiousness disorders	☐	☐	☐☐
Other: ______________________	☐	☐	☐☐

13) Has a brainwave current measurement (EEG, electroencephalography) been performed on the person concerned in the **last 3 months**?

Please cross the appropriate box.

☐ No ☐ Yes

14) Does the person concerned have one or more of the following **medical aids or devices**? This includes purchased or borrowed aids or devices.
Please first cross the box whether the person concerned has each aid or device.
If so, please continue to indicate if the person concerned used the aid or device in the **last 3 months**.

Aid or Device	No	Yes, he/she owns this	Yes, he/she has used it in the last 3 months
Wheelchair, multifunctional or nursing wheelchair	☐	☐	☐
Transfer aids, e.g hoist	☐	☐	☐
Standing aids, e.g. standing boards	☐	☐	☐
Walking aids, e.g. wheeled walker	☐	☐	☐
Bathing aids, e.g. shower couch or bathtub seats/mats	☐	☐	☐
Toilet aids, e.g. commode chair	☐	☐	☐
Hospital bed (height adjustable)	☐	☐	☐
Positioning aids, e.g. pillows, wedges	☐	☐	☐
Tracheostomy equipment	☐	☐	☐
Suction units	☐	☐	☐
Ventilator	☐	☐	☐
Inhalation devices	☐	☐	☐
Feeding tube, e.g. PEG-tube	☐	☐	☐
Feeding pump	☐	☐	☐
Communication devices e.g. technical, symbolic	☐	☐	☐
Orthoses or splints	☐	☐	☐
Aids for incontinence care	☐	☐	☐
Other: ______________________	☐	☐	☐
Other: ______________________	☐	☐	☐

15) Has the person concerned had any **physical therapy in the last 3 months**?
Home visits are also included.

Please cross the appropriate box.

- ☐ No → then continue with question **18)**
- ☐ Yes → then continue with question **16)**

16) If the person concerned has had any **physical therapy in the last 3 months**, please answer the following questions about the duration of the treatment.

Please cross and fill out the appropriate form.

Since when has the person concerned started physical therapy?

|_|_| Month |_|_|_|_| Year

On how many days per week does the person concerned have physical therapy?

on |_| days per week

On those days, how long did the physical therapy last on average?

on average |_|_| Hours and |_|_| Minutes

Was the physical therapy prescribed on a long-term basis? i.e. more than a usual case and **without** at least a three month therapy break?

No ☐ Yes ☐

17) **As a family member, relative or legal guardian**, how would you rate the following statements regarding the **physical therapy** of the person concerned?

Please cross the appropriate box.

**Through the Physical therapy….**	true	rather true	rather not true	not true
I was sufficiently informed.	☐	☐	☐	☐
I was given competent advice.	☐	☐	☐	☐
I received a guide on how to implement therapeutic or nursing procedures.	☐	☐	☐	☐
I was involved in the therapy process.	☐	☐	☐	☐
I was relieved mentally and physically.	☐	☐	☐	☐
the person concerned and I were enabled to partake in social activities.	☐	☐	☐	☐

18) Has the person concerned had any **occupational therapy in the last 3 months**?
Home visits are also included.

Please cross the appropriate box.

☐ No ⟶ then continue with question **21)**

☐ Yes ⟶ then continue with question 19**)**

19) If the person concerned has had any **occupational therapy in the last 3 months**, please answer the following questions about the duration of the treatment.

Please cross and fill out the appropriate form.

Since when has the person concerned started occupational therapy?

|_|_| |_|_|_|_|
Month Year

On how many days per week does the person concerned have occupational therapy?

on |_| Days per week

On those days, how long did the occupational therapy last on average?

on average |_|_| Hours and |_|_| Minutes

Was the occupational therapy prescribed on a long-term basis? i.e. more than a usual case and **without** at least a three month therapy break?

No ☐ Yes ☐

20) **As a family member, relative or legal guardian**, how would you rate the following statements regarding the **occupational therapy** of the person concerned?

Please cross and fill out the appropriate form.

**Through the occupational therapy…**	true	rather true	rather not true	not true
I was sufficiently informed.	☐	☐	☐	☐
I was given competent advice.	☐	☐	☐	☐
I received a guide on how to implement therapeutic or nursing procedures.	☐	☐	☐	☐
I was involved in the therapy process.	☐	☐	☐	☐
I was relieved mentally and physically.	☐	☐	☐	☐
the person concerned and I were enabled to partake in social activities.	☐	☐	☐	☐

21) Has the person concerned had any **voice, speech or language therapy in the last 3 months**? Home visits are also included.

Please cross the appropriate box.

☐ No ⟶ then continue with question **24)**

☐ Yes ⟶ then continue with question **22)**

22) If the person concerned has had any **voice, speech or language therapy in the last 3 months**, please answer the following questions about the duration of the treatment.

Please cross and fill out the appropriate form.

Since when has the person concerned started the voice, speech or language therapy?

|_|_| |_|_|_|_|
Month Year

On how many days per week does the person concerned have voice, speech or language therapy?

on |_| days per week

On those days, how long did the voice, speech or language therapy last on average?

on average |_|_| Hours and |_|_| Minutes

Was the voice, speech or language therapy prescribed on a long-term basis? i.e. more than a usual case and **without** at least a three month therapy break?

No ☐ Yes ☐

23) **As a family member, relative or legal guardian**, how would you rate the following statements regarding the **voice, speech or language therapy** of the person concerned?

Please cross and fill out the appropriate form.

**Through the voice, speech or language therapy…**	true	rather true	rather not true	not true
I was sufficiently informed.	☐	☐	☐	☐
I was given competent advice.	☐	☐	☐	☐
I received a guide on how to implement therapeutic or nursing procedures.	☐	☐	☐	☐
I was involved in the therapy process.	☐	☐	☐	☐
I was relieved mentally and physically.	☐	☐	☐	☐
the person concerned and I were enabled to partake in social activities.	☐	☐	☐	☐

24) Has the person concerned received any **professional nursing care in the last 3 months** (i.e. a registered nurse)? Home visits are also included.

Please cross the appropriate box.

☐ No → then continue with question **27)**

☐ Yes → then continue with question **25)**

25) If the person concerned has received any **professional nursing care in the last 3 months**, please answer the following questions about the duration of the treatment.

Please cross and fill out the appropriate form.

Since when has the person concerned received professional nursing care?

|_|_| |_|_|_|_|
Month Year

On how many days per week does the person concerned receive professional nursing care?

on |_| days per week

On those days, how long did the professional nursing care last on average?

on average |_|_| Hours and |_|_| Minutes

Does the professional nursing care take place on a long-term basis?

No ☐ Yes ☐

26) **As a family member, relative or legal guardian**, how would you rate the following statements regarding the **professional nursing care** received from the person concerned?

Please cross and fill out the appropriate form.

**Through the professional nursing care…**	true	rather true	rather not true	not true
I was sufficiently informed.	☐	☐	☐	☐
I was given competent advice.	☐	☐	☐	☐
I received a guide on how to implement therapeutic or nursing procedures.	☐	☐	☐	☐
I was involved in the therapy process.	☐	☐	☐	☐
I was relieved mentally and physically.	☐	☐	☐	☐
the person concerned and I were enabled to partake in social activities.	☐	☐	☐	☐

27) Has the person concerned had **any other therapies** (eg music therapy, animal-assisted therapy) **in the last 3 months**? Home visits are also included.

Please first enter the other therapies that the person concerned has had, and continue to indicate how often the person concerned has received these therapies.

Please cross and fill out the appropriate form.

☐ No

☐ Yes →

Other therapies	How often?
____________________	☐☐
____________________	☐☐
____________________	☐☐

28) Has the person concerned used the services of a **multidisciplinary rehabilitation team in the last 3 months**? Home visits are also included.

This refers to the use of various rehabilitative services (such as physiotherapy and occupational therapy), even if the nurses and therapists come from different institutions or external nurses and therapists are called in to strengthen the rehabilitation team.

Please cross the appropriate box.

- ☐ No → then continue with question **34)**
- ☐ Yes → then continue with question **33)**

29) **As a family member, relative or legal guardian**, how would you rate the following statements regarding the **cooperation between the rehabilitation teams?**

Please cross and fill out the appropriate form.

**The rehabilitation team…**	true	rather true	rather not true	not true	Don't know
follows a joint care and therapy plan.	☐	☐	☐	☐	☐
uses joint documentation.	☐	☐	☐	☐	☐
designs joint team and case discussions.	☐	☐	☐	☐	☐
works interdisciplinarily with the person concerned (e.g. treatment with two therapists of different disciplines)	☐	☐	☐	☐	☐
works according to a common care plan.	☐	☐	☐	☐	☐
Collaboration within the rehab team is not apparent to me.	☐	☐	☐	☐	☐

30) Does the household need to spend its own financial resources for the care of the person concerned? Co-payments of the statutory health insurance are also included.

Please cross and fill out the appropriate form.

☐ No

☐ Yes → If yes, how much in Euro per month is spent on average?

on average ☐☐☐☐ Euro per month

31) When was the person concerned born?

☐☐ Day ☐☐ Month ☐☐☐☐ Year

32) What is the gender of the person concerned?

☐ female

☐ male

33) What is the marital status of the person concerned?

married ☐

single ☐

divorced ☐

widowed ☐

34) Which is the highest school degree earned of the person concerned?

[to be defined according the countries' specific school system]	☐
	☐
	☐
No degree	☐
Other degree	________

35) What is the highest vocational qualification of the person concerned?

Vocational training	☐
Technical school/Technician/Master craftsmen school	☐
Engineering School/ Polytechnic school	☐
Higher education institution/ University degree	☐
No Qualification	☐
Other Qualification	________

36) Please estimate how difficult it was for you to complete the questionnaire.

very easy	☐
easy	☐
difficult	☐
very difficult	☐
impossible without help	☐

37) How much time did you need to complete the questionnaire?

☐☐ Minutes

38) If you wish, you can add comments on the content, level of difficulty, and clarity of the questionnaire or something similar.

If you have comments on individual questionnaire questions, please include the number of the specific question to which your comment refers to.

Please enter any comments here.

____
____
____
____
____
____
____

39) Please enter today's date.

Today's Date [|] [|] [| | |]
Day Month Year

Please double check that you have not missed a question!

Thank you for your support!
